# Patient Experience and Perception of First Language Usage in Healthcare: The Welsh Perspective

**DOI:** 10.1177/23743735261417165

**Published:** 2026-02-09

**Authors:** Maisie E. Edwards, Owen Bodger, Menna Brown, Llinos Roberts, Luke D. Roberts, Jeffrey S. Davies, Alwena H. Morgan

**Affiliations:** 1 Swansea University Medical School, 7759 Swansea University, Swansea, UK

**Keywords:** patient perspectives/narratives, bilingual care, language discordance, clinician–patient relationship, communication, inclusion, patient experience

## Abstract

Research shows that using a patient's preferred language is vital for effective healthcare communication. Consultations in a second language can lead to treatment delays and misdiagnoses. In Wales, while Welsh and English have equal status in the public sector, independent primary care providers like General Practices (GPs) are not fully bound by Welsh Language Standards (WLS), resulting in inconsistent bilingual provision. This mixed methods study combined focus groups and a survey of 361 Welsh speakers to explore awareness of WLS and experiences of bilingual GP care. Analysis revealed low awareness (27%) of the WLS and significant unmet language needs. 71% had never been offered a Welsh-language consultation and 57% with English-speaking GPs said they would feel more comfortable having Welsh-medium consultations. In high Welsh-speaking areas, 32% felt restricted by not being able to use their first language during GP appointments. There was strong support for recording language preference in health records. Findings highlight both the need and desire for Welsh-language provision in primary care, and the importance of policy changes to support an “active offer” approach.

## Introduction

Effective communication is vital to quality healthcare, yet the impact of the lack of bilingual provision has not been fully established. Language barriers can compromise patient healthcare^1^ especially when consultations occur in a patient's second language. This can lead to misinterpreted information, less treatment compliance, inadequate psychological support,^2^ and a lack of informed consent.^3^ Language discordance is also linked to delayed treatment, misdiagnosis, longer hospital stays, medication errors, poor management of chronic conditions, increased adverse events, and even death.^4–8^

Notably, the negative effects of language discordance have been documented in vulnerable groups, including children^9–11^ and older adults, particularly during dementia.^12^ Cultural differences between patients and healthcarers can also lead to less trust and contribute to adverse effects, even if they live in the same country.^13,14^ Much of this evidence comes from international research on bilingualism and minority languages in healthcare, but research specific to Welsh-medium healthcare is limited.

According to the 2021 Census, 29.1% of Wales’ population speak Welsh. While all Welsh speakers are considered bilingual (able to speak both Welsh and English), many prefer using Welsh, particularly in stressful or vulnerable situations.^15^ Importantly, health literacy is often lower in a person's second language,^16^ making language choice critical in healthcare. However, as a minority language, Welsh is not consistently available in healthcare settings, placing bilingual patients at a disadvantage.^17^ This is particularly concerning for vulnerable groups such as children who haven’t yet learned English, people with dementia who may lose their second language,^12^ and individuals with learning disabilities or mental health conditions who communicate more easily in their preferred language.^18^ For these groups, limited access to first language healthcare can compromise care delivery.^19^ For people in these groups with low health literacy, the lack of bilingual services may further impact their understanding, communication, and health outcomes.

Since 2018, all Health Boards in Wales must comply with and report on their compliance to the Welsh Language Standards (WLS), set by the Welsh Language Commissioner. However, these standards do not apply to independent healthcare providers, such as general practice (GP) surgeries, which are considered independent businesses.^20^ As a result, most GPs in Wales are not required to offer bilingual services or comply with WLS, and patients cannot demand Welsh-medium healthcare. This study explores Welsh speakers’ perceptions of Welsh-language use in primary care and assesses the demand/need for more bilingual rights and services in these settings.

## Methods

A staged mixed methods approach was used. Stage 1 involved a literature review and focus group exploration of Welsh language experiences in healthcare. Participants were recruited through purposive sampling, they were familiar with the context to help ensure that the questions reflected real experiences and priorities. They were invited by email to join online focus groups (using Zoom) held between 12/2021 and 01/2022. Participants provided written consent prior to taking part. Discussions were recorded and verbatim transcribed. Thematic analysis followed Braun and Clarke's (2006)^21^ method, supported by NVivo. Two researchers (AM, ME) independently familiarized themselves with the data, undertook initial coding whereby both researchers independently read, re-read, and coded raw data. Initial codes were discussed and overlapping codes were merged, unclear codes refined, and agreed definitions recorded to create one coding framework which was applied to the remaining transcript. When new codes emerged, they were discussed and agreed and transcripts reread. Codes were grouped into larger themes, discussed using a thematic map. Disagreements were resolved through discussion until a consensus was achieved. Candidate themes and transcripts were reviewed again to ensure no data were missed and all coded data aligned with final agreed themes. Finally, themes were named, and extracts selected. Themes informed the questionnaire design.

Stage 2 involved distributing an anonymous online questionnaire using Qualtrics XM. The questionnaire was promoted to Welsh speakers at Swansea University, through personal contacts and on social media (Twitter and Instagram). Inclusion criteria included being >18 years old, self-identify as being Welsh-speaking, able to give informed consent and internet access. Participants reviewed the participant information sheet before giving consent and commencing the survey. The 28-item survey covered demographics, language ability/preferences, and explored three themes: (1) Awareness and opinions of Welsh language rights in Healthcare; (2) Experiences of using Welsh in primary care, and (3) Perceived impact of Welsh on care quality (Table 1 and Appendix 1). Questions included a mix of closed answer and free-text responses. Initial analysis suggested regional variation, thus, in addition to data analysis of the whole cohort we also carried out regional subgroup analysis. Respondents were grouped by residence in areas with above or below 35% Welsh-speaking populations (“Higher” Welsh-speaking areas included: Gwynedd, Anglesey, Carmarthenshire, Ceredigion, Denbighshire, and Conwy). 35% was chosen based on data reported by the Office for National Statistics (ONS) (2021) of Welsh speakers in each Welsh county. When displayed as a percentage, the ONS data showed a clear visual separation at this level, with counties having either below 35% or above 35% Welsh speakers, making 35% a reasonable cut-off for sub-categorization. Quantitative data were analyzed using SPSS (version 28) using Chi-square analysis unless stated otherwise with significance being indicated by a 5% change. Free text responses underwent thematic analysis using pragmatic content analysis,^22,23^ informed by the results from Stages 1 and 2. For some, question frequency analysis was undertaken to identify common themes.

**Table 1.** Questionnaire Questions With Associated Theme.

Question number	Question theme	Question
11	1. Awareness and opinion of the Welsh language rights provision in healthcare	Are you aware that you do not have the right to demand primary healthcare through the medium of Welsh?
12		Were you aware that hospitals are expected to meet Welsh language standards set by the Welsh Language Commissioner (under the rights to Welsh language services) but they are not expected of primary healthcare organizations, eg, GP surgeries.
25		Do you feel that your need or right to use Welsh (or bilingually) during primary health care is taken seriously by places that provide primary health care?
26		If Welsh or another minority language is your language of preference, do you feel that it's right you have to sacrifice your preferred language in order to access healthcare services more quickly?
13	2.Personal experiences and opinions on the use of Welsh in primary care	Have you ever chosen a GP surgery based on the fact they have a Welsh speaking doctor?
14		If you usually have your GP consultations in English, would you feel more comfortable if they were held in Welsh/bilingual?
15		In which language do you tend to speak to your GP during clinical consultations, if they are Welsh speaking?
16		While making an appointment with the doctor, have you ever asked for a specific doctor because he/she speaks Welsh?
17		In which language would you feel most comfortable communicating with the receptionist in?
21		Have you ever had an offer from your GP to use Welsh as your language of choice during consultation?
22		Have you ever been in a situation where you've felt restricted by being only able to discuss in English with your GP?
18	3.Impact of Welsh on the quality of care	Do you think the use of Welsh language when receiving primary health care is an important part of your care?
19		Do you feel you benefit from receiving bilingual documents related to primary health care?
20		Would you benefit from verbal health care provision in Welsh?
23		If you were able to speak in Welsh and therefore feel more comfortable with your GP, do you believe that would have received a better healthcare service?
24		Would you be happy to note your language preference on your medical records?

## Results

### General Themes From the Focus Groups & Dual Interview

Eligible participants were Welsh-speaking adults aged over 18. The focus group included one male >60, two females aged 25-59, and one male <25. Due to two last-minute withdrawals, a second session was held as an extended dual interview with one male (>60) and one female (25-59) with no opportunity to re-organize additional focus groups due to time constraints of the project. These sessions explored participants’ healthcare experiences to help inform the questionnaire. Transcript analysis identified eight themes: “recognising the challenges facing the NHS,” “identification of vulnerable groups,” “patient's priorities,” “respect,” “responsibility of healthcare staff to ensure patient understanding,” “use of unofficial translators,” “impact of using a patients first language,” and “a change in perspectives over time” (Table 2).

**Table 2.** Themes and Sub-Themes Emerging From Focus Groups With Their Associated Quotations.

Emerging theme	Sub-theme	Example of associated quotation
Vulnerable groups of people	1.The Elderly	“Pobl uwchben wythdeg mlwydd oed, mae’r rhyddhad ma nhw’n cal pan ti’n mynd atyn nhw a phan ti’n siarad Cymrag da nhw, ti’n gweld e, ma fe jyst yn ‘diolch byth’, ma nhw’n gallu ymlacio. Mae’n rili bwerus. Fi’n cal hwna bron bob dydd. Fi’n cal ‘diolch byth’, ‘diolch byth’ bo chi’n siarad Cymraeg. A fi di cal pobl yn crio, yn dala’n ddwylo i, yn gweud ‘oh doctor’. Mae’n neud shwd gymaint o wahaniaeth iddyn nhw, wir, mae’n rili rili bwysig i rai bobol.” “*People over eighty years old, the relief they feel when you go up to them and when you speak in Welsh, you see it, it's just ‘thank goodness’, they can relax. It's really powerful. I get that almost every day. I get ‘thank goodness!, ‘thank goodness that you speak Welsh’. And I’ve had people crying, holding my hands, saying ‘oh doctor’. It makes such a difference to them, honestly, it's really important to some people.”* (Female, 40-49)
	2. Children	“plant a henoed ydy’r ddau categori pwysicaf dwi’n meddwl da ni angen y gwasanaeth i fod yn Gymraeg achos, ti isio nhw ddallt yn does? deall be sy’n mynd ymlaen. Ma nhw’n teimlo’n ‘vulnerable’, ma nhw’n teimlo ddim yn cant y cant. So, beth ma nhw angen ydy mynd at rhywun a nhw’n deud oce, paid a phoeni dyma beth sydd yn mynd ymlaen, dyna beth da ni angen, dyna beth ydy dynoliaeth, dyna pam da ni’n mynd at ddoctoriaid de, iddynt nhw ddaethon ni, oce newn ni sortio fo.” “*children and the elderly are the two most important categories that I think we need the service to be in Welsh, because, you want them to understand don’t you? to understand what's going on. They feel vulnerable, they don't feel 100 percent. So what they need is to go to someone and they say ok, don't worry this is what's going on, that's what we need, that's what humanity is, that's why we go to doctors, ok we'll sort it out.”* (Female, 20-29)
	3.People with mental health issues	“fi di cal cleifion ifanc, iau na fi, pobl yn ei ugeinie pan oni yn y Feddygfa yn dod ata i, a fi’n cofio un yn benodol, odd hi’n diodde o iselder ysbryd a bob tro odd hi’n dod mewn i’r ystafell odd hi’n gorjys, pob tro odd hi’n dod mewn odd hi’n dweud ‘fi’n dod ato ti Dr *** achos fi’n gallu siarad da ti, fi’n gallu siarad Cymrag. Fi ffaelu siarad da neb arall….mae’n effeithio pobl ifanc hefyd.” I've had young patients, younger than me, people in their twenties come to me when I was at the Surgery, and I remember one in particular, she was suffering from depression and every time she come into the room she was gorgeous, every time she came she’d say ‘I come to you Dr **** because I can talk to you, I can speak in Welsh . I can’t speak with anyone else’…. *it affects young people too.”* (Female, 40-49)
Effect of using a Patients’ first language	1.Comfortability	“Ma fe’n anodd achos ma lot mwy gyfforddus siarad yn y Gymraeg na beth yw e yn y Saesneg, yn enwedig os ma fe wrth glywed newyddion chi ddim isie clywed. Lot yn neisach clywed e yn iaith dy hun. Dim ond unwaith fi wedi gorfod gweld meddyg yn [lleoliad]. Odd neb yn siarad Cymrag lawr fan hyn.” “*It's difficult because it's a lot more comfortable to speak in Welsh than what it is in English, especially if it's news you don't want to hear. Much nicer to hear it in your own language. I have only had to see a doctor in [location] once. No one speaks Welsh down here.”* (Male, 20-29) “Ond mynd yn ôl at yr iechyd meddwl, pan ma pobl yn ‘vulnerable’, yn fregus, pan ma rhywun yn fregus ti’m isio gorfodi nhw i gorfod, wel sygynon nhw ddim yr egni i feddwl am yr eirfa mewn iaith arall de. Ti isio nhw fod mor gyffyrddus ag sy’n bosib, so bod nhw’n rili gallu agor i fyny a deud dyma beth sydd yn mynd ymlaen, ac os wyt ti’n gorfod meddwl am iaith a hyn hyn a llall, ti falle ddim yn mynd i gal y llun cryfa” “*But going back to mental health, when people are ‘vulnerable’, frail, when someone is fragile you don’t want to force them to have to, well they don't have the energy to think about the vocabulary in another language right. You want them to be as comfortable as possible, so that they can open up and say this is what's going on, and if you have to think about language and this and the other, you might not get the best picture”* (Female, 20-29)
	2.Expression of the patient	“Mae fel iaith wahanol, mae fel iaith ffurfiol da chi ond yn defnyddio pan da chi’n sal, ond wrth gwrs pan da chi’n sal, da chi ddim eisiau poeni lot am bethau so mae’n rhaid paratoi ar gyfer y sefyllfa na rhywsut.” “*It's like a different language, it's like a formal language you only use when you're ill, but of course when you're ill you don't want to worry too much about things like this, so you have to prepare for the situation no somehow.”* (Male, 70-79)
	3.Developing a rapport with a patient	“o bwynt gwneud rappour, o’r foment gynta, bydden i yn gweud ie, mae yn dylanwadu fi’n credu beth i chi’n meddwl o’r person o’ch blaen chi.” “*from the point of making rappour, from the first moment, I would say yes, i think it influences what you think of the person in front of you.”* (Female, 40-49)
A patient's priorities	1. Quality of healthcare	“ydy’n ni’n gorfod aberthu un am llall bron, de? dyle ni ddim gorfod, dyna eto ydy’r ideal senario, bod ni’m yn gorfod. A bod gynnon ni ffydd yn yr Cymry, bod gynnon ni ddoctoriaid, y gore, ar gael i ni. Ond dwi’n meddwl pe bydda yr hawliau yma yn dod i fyny, fysai o bosib, dwi’n trio meddwl o bob persbectif fama, fysa o bosib rhai teuluoedd, neu siaradwyr Cymraeg bron ddim yn hawlio achos fysa nhw isio’r doctor gora” “*do we have to sacrifice one for another almost, right? We shouldn't have to, that is again the ideal scenario, that we don’t have to. And that we have faith in the Welsh doctors, that we have the doctors, the best, available to us. But I think that if these rights come up, it would be possible, I'm trying to think from every perspective here, it could be that some families, or Welsh speakers don't claim the right because they want the best doctor”* (Female, 20-29)
	2.The importance of health	“…beth dwi’n trio pwysleisio ydy yn enwedig pan mae o’n dŵad i iechyd, iechyd sydd yn bwysig…” “*…what I'm trying to emphasize is especially when it comes to health, health is important…”* (Female, 20-29)
The responsibility of the health carer to ensure that the patient understands		"Fedri di sefyll fana a deud y sbiel efo’r geiriau mawr ma, grêt, ond efo doctoriaid mae o’n gyfrifoldeb arna nhw i sicrhau bod y patient yn deall beth sy’n mynd ymlaen er mwyn tynnu’r lefelau straen na, os ma nhw mewn cyfnod ofnadwy weithiau ma nhw’n rili… mae o’n bywyd neu marwolaeth i rai sefyllfaoedd, ti eisiau neud y patient sydd gynaf, ac er mwyn neud yn siŵr bod y patient sydd gyntaf, mae rhaid neud yn siŵr bod nhw’n deall bob elfen o’r sefyllfa a neud iddyn nhw teimlo’n hollol gyfforddus. I lot o bobl, yn amlwg yng Nghymru, Cymraeg ydy’r ffordd o neud hynny achos mamiaith ydy o, y gwreiddyn tyfna sydd gynno ni, so mae’n rhaid i ti gysylltu efo hyna er mwyn gwneud i bobl teimlo’n gartrefol” / “*You can stand there and say the spiele with these big words, great, but with doctors it is their responsibility to ensure that the patient understands what is going on in order to remove the stress levels, if they are in a terrible phase sometimes they are…* *it is life or death for some situations, you want to put the patient first, and in order to make sure that the patient comes first, you have to be sure they understand every element of the situation and make them feel completely comfortable. For a lot of people, obviously in Wales, Welsh is the way to do that because it's their mother tongue, we have deep root there, so you have to connect with that in order to make people feel at home”* (Female, 20-29)
Use of unofficial translators		“mae o wedi sylwi, mae o yn gorfod ailadrodd a chyfieithu beth mae’r doctoriaid ma’n ddeud i’r pobl sydd yn aros yma, y patient, mae o’n gorfod, ti’n gorfod mynd at lefel y patients yndwyt, os dydy nhw ddim yn deall yna wel beth ydy’r point.” “*he has noticed, he has to repeat and translate what the doctors are saying to the people who are staying here, the patient, he has to, you have to go to the level of the patients don’t you if they don't understand, well what's the point.”* (Female, 20-29)
Respect		“fi’n credu taw parch yw e. Nid falle, odi e’n neud i chi deimlo’n fwy cyfforddus yn syth, dim rili i fod yn hollol honest. Bydden i yn digon hapus, digon cyfforddus sa fe’n gweud ‘hello, how are you?’. Os yw e yn gweud “Bore da”, fi syth yn meddwl “Oh good egg”, oleia’ ma fe’n gwneud ymdrech. Ma fe’n parchu fi.” “*I think it's respect. Not that it makes you feel more comfortable straight away, not really to be completely honest. I'd be happy enough, comfortable enough if he said ‘hello, how are you?’. If he says “*Bore da (*Good morning)”, I immediately think “Oh good egg”, then he making an effort. He respects me.”* (Female, 40-49)
Recognition of the challenges facing the NHS	1. Fears about the practicalities of bilingual provision	“O’r safbwynt yn ochr gwladgarol, ma na fwy iddo fe na jyst byse fe’n neis i gael, achos ma na oblygiadau wahanol iddo fe hefyd. Ond da ni’n mynd i werthu’r syniad i neb, mae ‘na gwestiwn ymarferol” “*From the point of view of the patriotic side, there's more to it than it only beig nice to have, because there are different implications for it too. But we’re not going to sell that idea to anyone, there's a practical question”* (Female, 20-29)
	2.General lack of Healthcare workers, let alone ones that are bilingual	“Mae’r ateb delfrydol yn amlwg, dim jyst am y Gymraeg, ond does dim digon o doctors a nyrsys” “*The ideal solution is obvious, not just about the Welsh language, but there are not enough doctors and nurses”* (Male, 70-79)
	3.Problems with staff recruitment in addition to retaining staff	“trwy uniaethu a Chymru, mi fysai yn datrys broblem o recriwtio a phwysicach na hynny, cadw yr staff yng Nghymru” “*by identifying with Wales, it would solve the problem of recruitment and more important than that, keep the staff in Wales”* (Male, 70-79)
A change in perspectives over time	1. Perspectives of Welsh when young	“mae’r bobl ifanc yma yn mynd i ysgolion Cymraeg, oni’n siarad Saesneg yn ysgol, dwi’n dod o deulu Cymraeg, dwi’n siarad Cymraeg bob dydd, mae genai ffrindiau Cymraeg tu allan i ysgol digwydd bod, ond yn yr ysgol on i’n siarad Saesneg, a da chi’n gofyn wrthi fi pam…sgynai ddim syniad pam. Odd o yn rhywbeth, pwysau cymdeithasol, pwysau cymdeithas” “*these young people go to Welsh schools, they don't speak English at school, I come from a Welsh family, I speak Welsh every day, I happen to have Welsh friends outside of school, but at school I only speak English, and you ask me why…* *I have no idea why. It's something odd, social pressure, society's pressure” (Female, 30-39)*
	2. Perspectives of Welsh when older	“Mae fy ngwraig yn dod o ****, odd hi’n dweud bod **** yn lle Saesneg iawn nol yn y 50au, 60au, ond erbyn hyn mae’r hollol merched oedd yn gwrthod siarad Cymraeg pryd hynny, nhw sy’n rhedeg y gymdeithas Cymraeg lleol.” “My wife comes from ****, she says that **** was a very English place back in the 50 s, 60 s, but now, all the women who refused to speak Welsh at that time, they run the local Welsh society.” (Male 70-79)

### Questionnaire Results

#### Summary

Of the 377 submitted questionnaires, 361 were sufficiently complete (>50%) and included in the final dataset. The sample was predominantly female (76%, 272/357) and plurality aged 30-49 (45%) with 26%  < 30 and 29%  > 50. Respondents represented all 22 Welsh counties, with 44% from areas where over 35% of the population speak Welsh. Most were currently in work (73%), with students comprising a further 16%.

### Welsh Language Status of Participants

Although all participants were Welsh speakers, only 53% reported Welsh as their primary home language, with 18% using both Welsh and English. Around a quarter identified English as their main home language. This varied significantly with age (*P* = .004), with 63% of those >50 spoke Welsh at home, compared to just 38% of those <30.

Most respondents felt comfortable using Welsh: 77% verbally, 60% in writing, and 61% when reading. While comfort with reading and writing did not vary significantly by age, there was a significant association (*P* = .008) between age and spoken Welsh where 50% of those >50 preferred to express themselves verbally in Welsh.

Respondents from Welsh-speaking regions were more likely (*P* < .001) to have Welsh as their first language at home in comparison to areas with fewer Welsh speakers (91% vs 56%). A similar result was seen for people expressing themselves in Welsh, with 91% (verbal), 71% (writing), and 75% (reading) either preferring to use Welsh or being comfortable in both languages.

#### Results Theme 1—Awareness of the Lack of Right to Access to Bilingual Primary Healthcare in Wales

The 2022 *Mae gen i hawl* (“I have a right”) campaign aimed to raise awareness of the right of the public to Welsh-medium services. However, the WLS only apply to public entities, for example, health boards and not independent entities such as most GP surgeries-only around 9%, are managed by health boards (Welsh government statistics, 2019). Thus, there is likely confusion regarding who have to offer bilingual services. Survey responses confirmed low awareness, only 27% knew hospitals are required to meet WLS, and only 28% knew these rights do not apply to independent primary care providers. Awareness was significantly higher among those over 50, who were twice as likely to know their rights compared to under 30s. Awareness of language rights in hospitals in those >50 was 37%, compared to 19% for people <30s. The equivalent figures for language rights in independent primary care establishments were 36% and 17% respectively. No significant differences were found by region or gender. Overall, respondents felt that their need to use their first language is not taken seriously. Only 18% felt that this was given sufficient priority in primary care. Over half (53%) felt that it was unfair to “sacrifice” their preferred language to access faster care. Those living in Welsh-speaking regions were more likely to feel that their language needs were being taken seriously in primary healthcare (*P* = .008), relative to those from other regions (27% vs 14%).

#### Results Theme 2—Personal Experiences and Opinions on Using Welsh in Primary Care

After assessing public awareness of their rights to bilingual provision, we explored their personal experiences of bilingual healthcare. Overall, 44% (156/354) of respondents said they choose to speak Welsh with their GP. This figure increased to 60% (156/264) among those with a Welsh-speaking GP and a further 13% (34/264) choose to communicate bilingually. Use of Welsh varied by age (*P* = .001), with 82% (70/85) of over-50s choosing Welsh or bilingual communication, compared to 52% (32/62) of under-30s. In Welsh-speaking regions the average figure, across all ages, is even higher at 89% (119/133) and even in non-Welsh-speaking regions over half choose to use Welsh to some extent (54%, 71/131) if the GP could speak Welsh (*P* < .001). Where patients usually use English with their GP, 57% reported they would be more comfortable conversing in Welsh or using both languages. Even among the under 30s and in non-Welsh-speaking regions these figures were 49% and 42% respectively. While the consultation is the most critical part of engagement with primary care, an overwhelming majority (84%) felt more comfortable using Welsh (including bilingually) with reception staff.

Despite strong demand for the use of the Welsh, 71% (246/346) reported not being offered the option. Given the high numbers who use Welsh this suggests that the option is not always offered even where the GP is Welsh-speaking. However, Welsh provision is significantly more common in Welsh-speaking regions in comparison to non-Welsh-speaking regions (32% vs 14%, *P* < .001). As a result, 24% (83/345) reported feeling restricted by their inability to communicate in their preferred language, with this figure rising to 32% for respondents from Welsh-speaking areas (vs 18% elsewhere). Free-text responses highlighted these challenges particularly in the contexts of children (47%), older people (37%), mental health (10%), dementia (3%), and maternity care (3%). Language access is taken seriously by many, with 18% (63/354) actively requesting Welsh-speaking GPs and 20% (72/358) choosing a surgery that employ Welsh-speaking GPs. In Welsh-speaking regions, these figures rise to 25% (40/159) and 29% (46/159) respectively. Among those with a strong preference for Welsh during a consultation, 41% (64/156) have chosen a surgery to ensure this is possible.

#### Results Theme 3—Impact on Quality of Care

The questionnaire then explored the patients’ opinion on the impact of Welsh provision on quality of care. The majority of respondents (67%, 233/346) believed they would benefit from receiving verbal healthcare in Welsh. Older patients felt even more strongly (80%) but even among the under 30s the majority agreed (55%) (*P* < .001). The effect per region was similar, with 81% in Welsh-speaking regions believing they would benefit. Even in non-Welsh-speaking regions over a half (56%) shared this belief.

While 31% (105/337) felt that speaking Welsh with their GP would enhance treatment, 38% (129/337) disagreed. There was a stronger response to the use of bilingual documents, with 63% (220/350) reporting that this would improve their healthcare, rising to 69% in Welsh-speaking areas (*P* = .016). Twenty-two comments were left in free text boxes for this question, which revealed several themes. For comments relating to bilingual provision not adding value to their care there were 5 main themes: “no service improvement” (3 comments); “patients equally comfortable using English” (3 comments); “a lack of Welsh-speaking staff” (2 comments); “a restriction in services” (1 comment) and that “there might be a better service but not care” (1 comment). Conversely, 13 themes highlighted benefits of bilingual healthcare, such as, for “groups with increased requirement for the provision” (6 comments), “patient expression” (4 comments); “communication” (5 comments); “comfort” (2 comments); “improving the patient-clinician bond” (2 comments); one comment each regarding “understanding,” “support,” “relief,” “being judged on their English,” “the explanations of the patient being disregarded by English speaking clinicians,” “a reduction in language barrier,” “difficulties using translators” and “improved quality of care.” There was also one comment regarding being unsure of the impact of receiving treatment in Welsh.

It is clear from the responses that the Welsh language provision in primary care is an important issue. Overall, 73% of respondents felt that Welsh language services is an important aspect of their care, with 44% considering it *very* important. This increased to 89% in Welsh-speaking regions, but this opinion was also shared by a majority from non-Welsh areas (61%).

To facilitate an “active offer” of Welsh provision in primary care (rather than being requested) patients would need to make their preference clear on their medical records. We found almost complete support for this, 86% would be happy for their language preference to be recorded in their medical records, and a further 14% were unconcerned by this. Only one survey respondent was opposed to this.

### Thematic Analysis Results of Free Text Responses

Many respondents shared in-depth comments, overall, 298 comments were recorded, suggesting that many felt passionate about this topic. Nine key themes emerged: “Vulnerable groups,” “language choice,” “effect of using a patients first language,” “patients’ priorities,” “the presumption that everyone can and is happy to communicate in English,” “use of Unofficial translators,” “current services,” “challenges facing the NHS,” and “feelings of stigmatisation” (Table 3). Five of these themes overlapped with themes from the focus groups.

**Table 3.** Themes and Sub-Themes Emerging From Free Text Boxes in the Questionnaire With Examples of Their Associated Quotations.

Emerging theme	Sub-theme	Example of associated quotation
Vulnerable groups of people	1. Older people	“Er fy mod yn hollol rugl yn y ddwy iaith, pan arferwn ofalu am fy mam oedrannus, byddai hi wastad yn siarad gyda fi yn Gymraeg a minnau'n mynegi'r hyn ddwedai hi yn Saesneg wrth y doctor. Roedd Mam yn gallu siarad Saesneg yn iawn, wrth gwrs, ond wrth iddi heneiddio roedd hi'n llawer mwy cyfforddus yn Gymraeg.” “*Although I am completely fluent in both languages, when I used to look after my elderly mother, she would always speak to me in Welsh and I would express what she said in English to the doctor. Mum could speak English well, of course, but as she got older she was much more comfortable in Welsh.”* (Female, 50-59)
	2. Dementia	“Dwi'n credu mae'n delfrydol cael dewis ba iaith ac mae hyn yn arbennig o bwysig i blant a'r henoed. Mi allai meddwl fod rhywun gyda dementia yn cael gofal gwaeth wrth ddim cael y dewis Cymraeg.” “*I believe it is ideal to be able to choose which language and this is particularly important for children and the elderly. I could think that someone with dementia gets worse care when they don't have the Welsh option.”* (Female, 40-49)
	3.Children	“Iechyd fy mhlant, pan oeddent yn fach ac yn siarad Cymraeg yn bennaf ac angen mynegi eu hunain mewn ymgynghoriad.” “*My children's health, when they were small and spoke mainly Welsh and needed to express themselves in a consultation*.” (Female 40-49)
	4.Mental Health conditions	“Anodd trafod iechyd meddwl yn benodol yn Susneg” *"Difficult to discuss mental health specifically in English”* (Female 20-29)
	5. Pregnancy	“Having worked in maternity services and with elderly people in hospital I have seen numerous cases where patients felt less comfortable due to the unavalability of a welsh speaker to care for them.” (Female, 30-39)
	6.Menopause	“Mae heriau iechyd meddwl ac hefyd cyfnod y menopôs yn bendant yn rhai heriol i mi eu trafod yn fy ail iaith a byddwn wedi gwerthfawrogi gallu siarad am y rhain yn fy mamiaith.” “*The challenges of mental health and also the period of menopause are definitely challenging for me to discuss in my second language and I would have appreciated being able to talk about these in my mother tongue.”* (Female, 50-59)
Language Choice	1. Preference	“Byddai’n well gen i dderbyn gwasanaeth drwy’r Gymraeg ble bynnag bo hynny’n bosibl.” "*I would prefer to receive a service through the Welsh language wherever possible.”* (Male, 50-59)
	2. Availability of local care	“Erbyn heddiw, dydy dewis meddygfa na meddyg penodol (oherwydd ei bod yn siarad Cymraeg) ddim yn opsiwn. Rhaid derbyn y feddyfga mwyaf lleol a'r doctor sydd ar gael.” *"As of today, choosing a surgery or a specific doctor (because she speaks Welsh) is not an option. The most local pharmacy and doctor available must be accepted.”* (Female, 40-49)
	3. No option	“Yn y feddygfa dwi wedi fy nghofrestru ynddi (ym Mhontarddulais) does dim modd gofyn am feddyg penodol, felly hyd yn oed pe bai un sy'n siarad Cymraeg ni fyddai modd gofyn am apwyntiad gyda'r meddyg hwnnw!” “*In the surgery I am registered in (in Pontarddulais) it is not possible to ask for a specific doctor, so even if there was one who spoke Welsh it would not be possible to ask for an appointment with that doctor!”* (Female, 30-39)
	4. Successful active offer	“Dw i wedi ffeindio bod nifer o feddygon yn Abertawe yn siarad Cymraeg ac yn hapus i gynnig gwasanaeth / gyfathrebu yn Gymraeg. ‘Dyn ni wedi bod yn lwcus.” *“I have found that a number of doctors in Swansea speak Welsh and are happy to offer a service / communicate in Welsh. We've been lucky.”* (Female 30-39)
	5. The offer is a post code “lottery”	“O fi ddim yn gwybod fod ddim rhaid i cynnig gwasanaeth ddwy ieithog, oherwydd ma popeth yn ddwy ieithog yn ein syrjeri ni yn Caerfyrddin a dwi'n cael apwyntiadau Gymraeg pan dwi'n digwydd weld y doctorion sydd yn siarad Gymraeg.” “I *didn't know that you don't have to offer a bilingual service, because everything is bilingual in our surgeries in Carmarthen and I get appointments in Welsh when I happen to see the doctors who speak Welsh”* (Female, 30-39)
	6. No offer	“Does dim cynnig rhagweithiol o Gymraeg yng Ngofal Sylfaenol, ymhellach mae gofyn am wasanaeth yn y Gymraeg yn cael ei hystyried yn rhwystr.” “*There is no proactive offer of Welsh in Primary Care, furthermore, asking for a service in Welsh is considered an obstacle.”* (Female, 30-39)
	7. Awkwardness / lack of confidence in requesting preferred language	“mae'n ddgon anodd cael apwyntiad yn y lle cyntaf, heb feddwl am geisio cael apwyntiad gyda'r meddyg sy'n siarad Cymraeg. Mae cyn lleied o opsiynau ar gael o ran gweld meddyg, heb son am feddyg sy'n gallu siarad Cymraeg. Mae staff y derbynfa yn digon swrth fel y mae - pe byddwn yn siarad Cymraeg gyda nhw, duw a wyr beth fyddai'r ymateb…” “*…it is quite difficult to get an appointment in the first place, without thinking about trying to get an appointment with the Welsh speaking doctor. There are so few options available when it comes to seeing a doctor, let alone a doctor who can speak Welsh. The reception staff are blunt enough as it is - if we spoke Welsh to them, god knows what the response would be…”* (Female, 50-59)
	8. Training for designation of Welsh speaking patients to Welsh speaking healthcarers	“Mae llawer o gyfrifoldeb ar weithwyr derbynfa i wneud penderfyniadau ar ran claf mewn ffordd ac mae angen eu haddysgu hwy o bwysigrwydd rhoi claf sy'n siarad Cymraeg i weld meddyg Cymraeg os yw hynny'n bosibl heb sôn am geisio sichrau bod meddygon teulu sy'n siarad Cymraeg ym mhob meddygfa a phwysigrwydd y drefn hyfforddi meddygon ar gyfer hynny.” “*Reception workers have a lot of responsibility to make decisions on behalf of a patient in a way and they need to be educated on the importance of giving a Welsh speaking patient to see a Welsh doctor if that is possible not to mention trying to ensure that there are family doctors who speak Welsh in every surgery and the importance of the doctors’ training system for that.”* (Female, 40-49)
	9. Some are happier to communicate in English	“I have never had an issue with speaking in English instead of Welsh in a clinical setting. Its more suitable for me since I understand the clinical terms better in English.” (Male, 18-20)
	10. Having to “sacrifice” one's language choice to gain access to care	“Ond rwy'n teimlo'n gryf bod hi'n anghywir fod rhaid i berson sy'n defnyddio Cymraeg (neu iaith leiafrifol arall) fel iaith gyntaf aberthu eu hiaith ddewisol er mwyn cyrchu gwasanaeth gofal iechyd yn gynt.” “*But I feel strongly that it is wrong that a person who uses Welsh (or another minority language) as a first language must sacrifice their chosen language in order to access a healthcare service sooner.”* (Male, 20-29)
Effect of using a Patients’ first language	1. Comfortability	“Being able to speak Welsh at Healthcare appointments, esp. intimate examinations makes me feel much more relaxed and at ease.” (Female, 40-49)
	2. Easier to use generally	“Heb os, mae'r gallu i ddefnyddio'r Gymraeg yn gwneud fy mhrofiad i fel claf yn haws ac yn fwy hwylus. Dyna'r iaith rwy'n teimlo fwyaf cartrefol ynddi. Er fy mod yn medru'r Saesneg, nid dyna fy iaith gyntaf na fy iaith emosiynol.” “*Without a doubt, the ability to use the Welsh language makes my experience as a patient easier and more convenient. It's the language I feel most at home in. Although I know English, it is not my first language or my emotional language.”* (Female, 30-39)
	3. Easier when explaining a particular problem	“Rwyf wastad wedi teimlo llawer mwy cartrefol pan yn siarad gyda doctor neu nyrs sy'n siarad Cymraeg. Dwi'n gallu peidio â phoeni am sut i esbonio fy hunan yn Saesneg a chanolbwyntio ar esbonio beth sy'n bod yn fy ngeiriau naturiol fy hun.” “*I have always felt much more at home when talking to a doctor or nurse who speaks Welsh. I can stop worrying about how to explain myself in English and focus on explaining what's wrong in my own natural words.”* (Female, 40-49)
	4. Relief	“pan rwyf yn dod ar draws rhai sy’n siarad Cymraeg mae’n gysur mawr, yn rhyddhad, ac yn brofiad hollol wahanol” “*when I come across those who speak Welsh it is a great comfort, a relief, and a completely different experience”* (Female, 50-59)
	5. Promoting a more natural behaviour/response through first-language communication	“Rwyf wedi sylwi ei bod yn od iawn cael ymwelydd iechyd sy'n siarad Saesneg i deulu Cymraeg iaith gyntaf. Mae hi'n ceisio ymdrin ȃ fy mhlant yn Saesneg ond nid ydynt yn ymateb yn eu ffordd arferol gan taw Cymraeg yw eu hiaith gyntaf.” “*I have noticed that it is very odd to have a health visitor who speaks English to a first language Welsh family. She tries to deal with my children in English but they don't respond in their usual way as Welsh is their first language”* (Female, 30-39)
	6. Better expression/ communication	“Pan oeddwn i'n trio esbonio sut oedd poen yn teimlo. Dwi'n teimlo mod i'n gallu esbonio dipyn gwell yn Gymraeg.” “*When I was trying to explain what pain felt like. I feel that I can explain much better in Welsh.”* (Female, 40-49)
	7. Effect of mispronouncing Welsh names / Lack of cultural respect	“…oedd y nyrs yn trio ei ddihuno yn yr uned gofal dwys ond yn methu ynganu ei enw (Aurwel, sy'n cael ei ynganu'n ‘Eirwel’, ond roedd yr hyn oedd hi'n trio dweud mwy fel ‘awe-wil’, a byddai fy nhad ddim o reidrwydd wedi adnabod hyn yn y stad roedd e ynddo). Rwy'n deall bod yr enw yn un anodd, ond doedd dim ymdrech i geisio deall yr ynganiad cywir.” “*…the nurse was trying to wake him in the intensive care unit but couldn't pronounce his name (Aurwel, which is pronounced ‘Eirwel’, but what she was trying to say was more like ‘awe-wil’, and my father would not necessarily have recognized this in the estate he was in). I understand that the name is difficult, but there was no effort to try to understand the correct pronunciation.”* (Female, 40-49)
	8. Reducing stress for the patient	“Mae gorfod siarad Saesneg yn ychwanegu at sefyllfa sy’n llawn straen be’ bynnag, gwneud pethe’n llawer anoddach. Mae’n anodd iawn mynd â fy mhlant at feddyg di-Gymraeg, ddim yn teimlo fod y plant yn cael yr un cysylltiad. Roedd un yn dioddef o orbryder, ac roedd hi’n berson gwahanol gyda’r meddyg Cymraeg o’i gymharu ag un Saesneg” “*Having to speak English adds to a stressful situation, anyway, making things much more difficult. It is very difficult to take my children to a non-Welsh doctor, I don't feel that the children get the same connection. One suffered from anxiety, and she was a different person with the Welsh doctor compared to an English one”* (Female, 40-49)
A patient's priorities	1. Quality of care	“Safon y gofal iechyd yn bwysicach na pa iaith sy’n cael ei siarad er byddwn yn fwy cyfforddus yn Gymraeg” “*The standard of healthcare is more important than what language is spoken although I would be more comfortable in Welsh”* (Female, 30-39)
	2. Waiting times	“Byddai'n well gen i dderbyn y gwasanaeth yn gynt a'i fod o safon uchel. Byddai'n well gen i dderbyn gwasanaeth Saesneg yn gyflym nag aros yn hirach am wasanaeth Cymraeg.” “*I would prefer to receive the service sooner and for it to be of a high standard. I would rather receive an English service quickly than wait longer for a Welsh service.”* (Female, 50-59)
	3. Best specialist for care	“Well gweld yr arbennigwr gora dim ots pa iaith. Tydi saesneg rhai ddim yn gret!!” “*Better to see the best specialist no matter which language. Some people's English is not great!!”* (Female, 40-49)
	4. Public spending	“Yn personol ar ol teithio'r byd rwyn meddwl fod ni'n hynod o lwcus i gael yr NHS, yn enwedig i gymaru gwledydd eraill. Fyddai'n hapusach i sicirhau arian hyr tymor i ffocws ar triniaeth rhydd,dros ffocws ar yr iaith sydd yn cael ei cynnal mewn” “*Personally after traveling the world I think we are extremely lucky to have the NHS, especially compared to other countries. I’d be happier if money was secured in the long-term to focus on free treatment, over a focus on the language that is maintained in”* (Female, 30-39)
The presumption that everyone can and is happy to communicate in English	1.Verbal	“Yn teimlo mor rhwystredig yn ein Meddygfa am fod agwedd llawer sy'n cael eu cyflogi yno mor negyddol ac anwybodus tuag at yr Iaith Gymraeg. Dim ymdrech gan lawer ohonynt i GEISIO dysgu'r Gymraeg a llawer wedi dod dros Glawdd Offa am fod tai yn rhatach yng Nghymru a byw yma, mewn gwlad wahanol, yng nghefngwlad, ar eu telerau hwy eu hunain ac agwedd fod pawb ymhobman yn gallu siarad Saesneg” “*Feeling so frustrated in our Surgery because the attitude of many who are employed there is so negative and ignorant towards the Welsh Language. Many of them make no effort to TRY to learn the Welsh language and many have come over Offa's Dyke because houses cheaper in Wales and live here, in a different country, in the country, on their own terms and with the attitude that everyone everywhere can speak English”* (Female, 70-79)
	2.Documentation	“Mae'r meddygon sydd yn siarad Cymraeg yn cymryd yn ganiataol ein bod eisiau dogfenwaith Saesneg, a ddim hyd yn oed yn meddwl argraffu fersiwn Cymraeg. Mae rhai meddygon yn wych ac yn darparu'n Gymraeg ond mae'r cyfan ar hap.” “*The doctors who speak Welsh assume that we want English documentation, and don't even think of printing a Welsh version. Some doctors are great and provide in Welsh but it's all random.”* (Female, 40-49)
	3. Taking it for granted that English is preferred	“Doedd dim ffurflenni dwyieithog gan fy neintydd er mwyn i fi gofrestru fy mhlant. Gofynnais iddyn nhw ddarparu ffurflen yn Ddwyieithog. Wnaethon nhw ddim ac erbyn hyn (2 flynedd ar ol aros - oherwydd y pandemig) fe lenwais y ffurflen Saesneg oherwydd mae angen i fy mhlant weld deintydd. Ond roeddwn i’n siomedig a rhwystredig iawn. Mae fy neintydd a sawl un arall yn y practis yn siarad Cymraeg hefyd. Mae pobl ddiGymraeg yn cymryd y pethau yma yn ganiataol.” “*There were no bilingual forms from my dentist for me to register my children. I asked them to provide a form in Bilingual. They didn't and now (2 years after waiting - due to the pandemic) I filled in the English form because my children need to see a dentist. But I was very disappointed and frustrated. My dentist and several others in the practice also speak Welsh. Non-Welsh speakers take these things for granted.”* (Female, 40-49)
	4. English is the “default”	*“*Mae'r Saesneg wastad ‘di bod yn y “default” gyda fy noctoriaid.” “*English has always been the “default” with my doctors.”* (Male, 30-39)
	5. Lack of awareness by English staff working in Wales	“Yn teimlo mor rhwystredig yn ein Meddygfa am fod agwedd llawer sy'n cael eu cyflogi yno mor negyddol ac anwybodus tuag at yr Iaith Gymraeg. Dim ymdrech gan lawer ohonynt i GEISIO dysgu'r Gymraeg a llawer wedi dod dros Glawdd Offa am fod tai yn rhatach yng Nghymru a byw yma, mewn gwlad wahanol,yng nghefngwlad, ar eu telerau hwy eu hunain ac agwedd fod pawb ymhobman yn gallu siarad Saesneg.” “*Feeling so frustrated in our Surgery because the attitude of many who are employed there is so negative and ignorant towards the Welsh Language. Many of them make no effort to TRY to learn the Welsh language and many have come over Offa's Dyke because houses cheaper in Wales and live here, in a different country, in the hinterland, on their own terms and with the attitude that everyone everywhere can speak English” (Female, 70-79)*
Current Services	1.Lack of the “Welsh offer”	“Nid oes dewis am y Gymraeg wedi bod inni ers yr wyf gyda'm Meddyg Teulu presennol ers yn agos i 50 mlynedd” “*We have had no choice about the Welsh language since I have been with my current GP for nearly 50 years”* (Female, 70-79)
	2. Promoting Welsh services	“Does neb byth yn cynnig gwasanaeth Cymraeg. Weithiau mae e ar gael ond ddim yn cael ei hyrwyddo.” “*Nobody ever offers a Welsh language service. Sometimes it's available but not promoted.”* (Female, 40-49)
	3. Restricted availability	“Not all GP's speak Welsh in my surgery, so the service will be more restricted.” (Female, 40-49)
	4. Welsh correspondence	“I used to work in my local GP surgery. Many patients notes were highlighted stating they wanted all correspondences in Welsh. This was provided as much as possible.” (Female, 40-49)
	5. Lack of confidence by staff	“Ar ôl iddo ddechrau gwella bu mewn ward arall lle roedd nyrs yn gofalu amdano oedd wedi bod i'r ysgol uwchradd leol (“blynyddoedd yn ôl”), a ddim â'r hyder i ddweud yr un gair wrtho yn y Gymraeg. Cymraeg digon tafodiaethol sydd gan fy nhad, o'r un ardal â hi, a byddai hyd yn oed sgwrs yn llawn ‘Wenglish’ wedi codi ei galon tipyn…. dangos y pwysigrwydd o hyfforddi staff, o ynganu ar un pegwn, i'r hyder i'w defnyddio heb poeni am gywirdeb, ar y arall.” “*After he started to recover, he was in another ward where he was looked after by a nurse who had been to the local secondary school (“years ago”) and didn't have the confidence to say a single word to him in Welsh. My father has quite a local dialect, from the same area as her, and even a conversation full of ‘Wenglish’ would have cheered him up quite a bit… show the importance of training staff, from pronunciation on one extreme, to the confidence to use them without worrying about accuracy, on the other.”* (Female, 40-49)
	6. Lack of request	“With my English accent, it's often assumed that I don’t speak Welsh, and I’m seldom asked if I do.” (Female, 60-69)
	8. Difficulties recognising/identifying Welsh speaking staff	“My GP surgery does offer to communicate in Welsh but it isn't clear to me as to which Drs, if any, speak Welsh.” (Female, 20-29)
	9. Unequal/improper implementation of Welsh provision	“Mae delio gyda'r ysbysty cyfagos, Ysbyty Gwynedd, ar y llaw arall yn fater gwahanol. Er fod sgyrsiau ac ymgynghoriadau meddygol yn digwydd yn Gymraeg os yw'r ddau ohonom yn siaradwyr Cymraeg, y Saesneg yw'r iaith ddiofyn. Prin iawn y caiff apwyntiadau ac ymgynghoriadau Cymraeg eu cynnig, a hyd yn oed ble mae cofnod o ddewis iaith yn bodoli (ynddo'i hun yn beth prin) hap a damwain yw derbyn gwasanaeth Cymraeg.” “*Dealing with the neighboring hospital, Ysbyty Gwynedd, on the other hand, is a different matter. Although conversations and medical consultations take place in Welsh if we are both Welsh speakers, English is the default language. Welsh appointments and consultations are very rarely offered, and even where a record of language choice exists (in itself a rare thing) receiving a Welsh service is by chance.”* (Male, 40-49)
	10. Prioritisation of foreign languages over Welsh in Wales	“Dwi ddim yn gweld unrhyw reswm dros ieithoedd lleiafrifol erall yn cael yr un driniaeth (yng Nghymru). Y gwir yw, weithiau, mae ieithoedd tramor yn cael mwy o bwyslais gan sefydliadau mawr na'r Gymraeg, sy'n drist iawn.” “*I don't see any reason for other minority languages getting the same treatment (in Wales). The truth is, sometimes, foreign languages are given more emphasis by large organisations than Welsh, which is very sad.” (*Male, 30-39)
Use of Unofficial translators	1. Parents translating for their children	“Yr un peth a mynd ar plant i'r meddyg, mae disgwyl cyfieithu popeth nôl ac ymlaen a fydde nhw yn medru esbonio llawer haws yn Gymraeg” “*The same as taking children to the doctor, everything is expected to be translated back and forth and they would be able to explain much more easily in Welsh”* (Female, 30-39)
	2. Adult children translating for their parents/grandparents	“Pan oedd Mamgu yn yr ysbyty roedd ddim yn medru deall yr arbenigwyr a dim syniad gyda hi beth oedd yn mynd ymlaen gan nad oeddent yn siarad Cymraeg.” *“When my grandmother was in hospital, she couldn't understand the specialists and she had no idea what was going on as they didn't speak Welsh.”* (Female, 30-39)
	3. Effect of translation on the patient	“Roedd y doctor yn cadw holi pam o ni yn siarad Cymraeg i fy mhlentyn, wrth i mi gyfieithu beth oedd y doctor yn gofyn iddo. Er mae'r mab yn deall lot fawr o'r Saesneg erbyn nawr, fe aeth yn swil i gyd, ond unwiath i mi gyfieithu iddo fe, roedd yn ateb yn iawn.” “*The doctor kept asking why we spoke Welsh to my child, as I translated what the doctor was asking him. Although the son understands a lot of English by now, he became all shy, but once I translated for him, he answered with no problem.”* (Female, 30-39)
	4. Effect on the unofficial translator / Responsibility ensuring understanding while translating	“Pan yn mynd ar plant i'r meddyg mae'n annodd iawn gan fod yn ddisgwyliedig i mi gyfieithu iddynt ddeall” “*When taking children to the doctor it is very difficult as I am expected to translate for them to understand”* (Female, 30-39)
	5. 'Internal' translating	“Sometimes you can’t explain properly how you feel in english when your constantly trying to translate” (Female, 50-59)
	6. Feelings of discomfort/strain on relative	“…Roedd rhaid imi gyfieithu i fy mab ac roeddwn yn teimlo'n anghyffyrddus…” “*…I had to translate for my son and I felt uncomfortable…”* (Female, 40-49)
	7. Translation undermining the severity of a situation	“Mae trafod anhwylder yn gallu bod yn brofiad emosiynol. Mae gallu mynegi yr heriau, y boen, y pryder yn fy ail iaith yn tynnu’r emosiwn ac yn gallu mynegi sefyllfa sy’n llai difrifol na’r realiti. Mae methu canfod y geiriau iawn dan straen yn gallu profi’n rhwystredig hefyd.” “*Discussing a disorder can be an emotional experience. Being able to express the challenges, the pain, the anxiety in my second language removes the emotion and can express a situation that is less serious than the reality. Not being able to find the right words under stress can also prove frustrating.”* (Female, 50-59)
	8. Risks of translation	“… Teimlo bysa hyn yn gallu creu risg i blant mewn perygl petai nhw ddim yn gallu cyfathrebu gyda’r Ymwelydd Iechyd os oedd problem adref.” “*…* *I feel that this could create a risk for children at risk if they could not communicate with the Health Visitor if there was a problem at home.”* (Female, 50-59)
Recognition of the challenges facing the NHS	1. General pressures on GPs	“mae hi mor anodd cael apwyntiad meddyg a sylweddoli fod na bwysau gwaith ar y doctoriaid felly rwyf yn derbyn pwy bynnag sydd ar gael ond yn falch iawn os na rywun sy'n siarad Cymraeg dwi'n cael.” “*it is so difficult to get a doctor's appointment and realize that there are work pressures on the doctors so I accept whoever is available but I am very pleased if I get someone who speaks Welsh.”* (Female, 50-59)
	2. Long waiting lists	“dwi'n poeni mwy am hyd waiting lists a pa mor anodd yw hi i gael apwyntiad gyda'r GP ddim pa iaith byddai'n siarad pan dwi yna.” “*I'm more worried about the length of waiting lists and how difficult it is to get an appointment with the GP not what language I would speak when I'm there*” (Female, 30-39)
	3. Practicalities of bilingual provision	“Mae'n gymleth. Dwi'n gweithio yn y maes iechyd felly dwi'n deall byddai hyn yn anodd i meddygfeydd.” “*It's complicated. I work in the health field so I understand this would be difficult for surgeries”* (Female, 40-49)
	4. Situational awareness	“Petai sefyllfa'r GIG yn well, bydden i'n llawer fwy llafar am yr angen i sicrhau gofal iechyd sylfaenol yn Gymraeg.” “*If the NHS situation were better, I would be much more vocal about the need to ensure basic health care in Welsh.”* (Female, 30-39)
	5. General lack of healthcare workers	“Tydi o ddim yn iawn ond nid yw’n cael i ystyried yn bwysig gan fod rhan fwyaf yn gallu siarad saesneg a gyda prinder staff mae unrhyw wasanaeth yn well na dim” “*It's not right but it isn’t considered important as most can speak English and with a shortage of staff any service is better than none*” (Female, 30-39)
	6. General lack of Welsh speaking doctors	“Mae Cymru yn bryn o feddygon teulu fel y mae hi, ac mae canran sylweddol o feddygon teulu dan hyfforddiant o wledydd tramor. Mae'n bwysig ein bod yn ceisio hyfforddi mwy o feddygon teulu Cymraeg eu hiaith” “*Wales is lacking family doctors as it is, and a significant percentage of family doctors in training are from foreign countries. It is important that we try to train more Welsh-speaking GPs”* (Male, 60-69)
	7. A lack of Welsh speaking healthcare workers in specialist fields	“Wedi cael profiad o driniaethau a gofal ysbytai a gwasanaethau canser yn ddiweddar a phrin yw’r arbenigwyr Cymraeg eu hiaith; pan rwyf yn dod ar draws rhai sy’n siarad Cymraeg mae’n gysur mawr, yn rhyddhad, ac yn brofiad hollol wahanol…” “*I’ve reccently had experience of treatments and care in hospitals and cancer services and there are few Welsh-speaking specialists; when I come across those who speak Welsh it is a great comfort, a relief, and a completely different experience…”* (Female, 50-59)
	8. Problems with recruiting staff generally	“Mae recriwtio siaradwyr Cymraeg i weithio yng ngofal sylfaenol hefyd yn broblem yn enwedig mewn ardaloedd cefn gwlad. Rhaid gwneud fwy i hyrwyddo gyrfaoedd iechyd yn ein hysgolion a cholegau i ddatrys y broblem yma.” “*Recruiting Welsh speakers to work in primary care is also a problem, especially in rural areas. More must be done to promote health careers in our schools and colleges to solve this problem.”* (Female, 50-59)
	9. Problems with recruiting bilingual staff	“Teimlaf beth cydymdeimlad gyda fy mhractis gan eu bod wedi methu cyflogi meddygon Cymraeg eu hiaith gan bod dim un wedi trio am y swydd. Angen sicrhau bod meddygon sy’n siarad Cymraeg yn gallu aros yng Nghymru ar ol cymwyso.” “*I feel some sympathy with my practice as they have failed to employ Welsh speaking doctors as none have tried for the job. Need to ensure that doctors who speak Welsh can stay in Wales after qualifying*.” (Female, 60-69)
	10. Geographical challenges	“While living in Gwynedd, comparing Swansea and Gwynedd is very different where Welsh is the primary language used in GP's compared to in Swansea, personally it depends on the area that you're in weather the receptionists and GP's speak welsh. But all GP's should have the ability to speak welsh for patients” (Male, 20-29)
	11.The understanding of the bilingual public	“Ond deallaf bod meddygon Cymraeg yn brin ac felly byddwn yn fodlon cael ymgynghoriad Saesneg pe bai rhaid. Ar ddiwedd y dydd mae iechyd yn bwysicach.” “*I understand that Welsh speaking doctors are rare and therefore I would be willing to have an English consultation if necessary. At the end of the day health is more important.”* (Female, 40-49)
	12. Lack of leadership and policy implementation in primary care	“Mae angen prosesau ac arweiniad clir a bathodynnau iaith gwaith a chynnig rhagweithiol. Mae'r cyfan mewn lle ond angen cael ei weithredu yn gyson ac o ddifri.” “*We need clear processes and guidance and language badges for work and a proactive offer. It's all in place but needs to be implemented consistently and seriously.”* (Female, 40-49)
	13. Lack of consistency	“Mae cymaint o amrywiaeth o sut mae'r Gymraeg yn cael ei ddarparu o feddygfa i feddygfa.” “*There is such a variety of how Welsh is provided from surgery to surgery.”* (Female, 40-49)
	14. Ensuring quality of bilingual language skills	“bydd angen cael cryn gynnydd o ran recriwtio Cymry Cymraeg i’r proffesiwn a sicrhau bod eu sgiliau iaith yn rhagorol yn y ddwy iaith er mwyn osgoi camddealltwriaeth yn ystod ymgynghoriad a allai achosi dirywiad o ran ansawdd.” “*there will need to be considerable progress in terms of recruiting Welsh speakers into the profession and ensuring that their language skills are excellent in both languages in order to avoid misunderstandings during consultation which could cause a decline in quality.”* (Male, 50-59)
	15. Challenges of keeping bilingual notes	“Gan ystyried y ffaith bod angen i feddygon eraill (rhai ohonynt yn ddi-Gymraeg) yn gorfod darllen nodiadau, byddwn am weld system ble byddai modd i unrhyw feddyg fedru darllen y nodiadau yn y naill iaith.” “*Considering the fact that other doctors (some of them non-Welsh speaking) need to read notes, I would like to see a system where it would be possible for any doctor to be able to read the notes in either language.”* (Male, 50-59)
Feelings of stigmatisation	1. Belittling	“…pam ddylai fod angen imi gyfieithu iddo fo, roeddwn yn gweld bod y sefyllfa yn ei fychanu fo a'i gyflwr rywsut…” “*…why should I have to translate for him, I felt as if the situation somehow belittled him and his condition…”* (Female, 40-49)
	2. Feeling daft	“Rydw i’n aml wedi cael fy nal methu cofio beth yw term Saesneg am bethe ee crachen, briw. Hynny’n gwneud i mi deimlon wirion, ac yn ychwanegu at straen y sefyllfa” “*I've often been unable to remember what the English term is for eg scab, sore. That makes me feel daft, and adds to the stress of the situation” (*Female, 40-49)
	3. Injustice of not being able to your first language in your own country	“Dyw hi ddim yn iawn mod i'n gorfod defnyddio ail iaith yn fy ngwlad fy hun…” “*It's not right that I have to use a second language in my own country…”* (Female, 30-39)
	4. Perceived stigma causing the want to complain	“Weithiau mae'n teimlo ein bod yn aberthu fy nheimladau i sicrhau fy mod i neu aelodau'r teulu yn cael gwasanaeth gofal sylfaenol, e.e. apwyntiad. Serch hynny, fel allen i ddadlau bod angen i mi gwyno er mwyn i'r sefyllfa gael ei drin o ddifrif ac i bethau ddechrau gwella.” “*Sometimes it feels like we’re sacrificing my feelings to ensure that I or family members get primary care service, eg an appointment. Nevertheless, I could argue that I need to complain for the situation to be treated seriously and for things to start to improve.”* (Female, 30-39)
	5. The stigma of Welsh being a minority language in Wales	“Mae gennyf broblem gyda'r cwestiwn sy'n grwpio Cymraeg gyda unrhyw ‘ iaith leiafrifol arall’?” “*I have a problem with the question that groups Welsh with any ‘other minority language'?”* (Female, 50-59)
	6. Questioned for speaking Welsh	“Roedd y doctor yn cadw holi pam o ni yn siarad Cymraeg i fy mhlentyn, wrth i mi gyfieithu beth oedd y doctor yn gofyn iddo… wnes i adael y feddygfa mae sioc bron bod y doctor yma yn cadw fy nghwestiynnu dros fy newis i o iaith gyda fy mab yng NGHYMRU.” “*The doctor kept asking why I spoke Welsh to my child, as I translated what the doctor was asking him…* *I left the surgery in shock that this doctor kept questioning me over my choice of language with my son in WALES.”* (Female, 30-39)
	7. Ignorance of Staff	“Cefais brofiad pan oedd aelod or teulu yn glaf mewnol. Cafodd ei chyhuddo o fod yn gymsyglyd ei meddwl am ei fod yn siarad cymraeg gan aelod o’r staff. Sefyllfa warthus ofnadwy !!!!” “*I had an experience when a family member was an inpatient. She was accused by a member of staff of being confused because she spoke Welsh. A terrible disgraceful situation!!!!”* (Female, 60-69)
	8. A need for more staff training on the importance of Welsh for bilingual speakers to reduce stigma	“…Mae llawer o gyfrifoldeb ar weithwyr derbynfa i wneud penderfyniadau ar ran claf mewn ffordd ac mae angen eu haddysgu hwy o bwysigrwydd rhoi claf sy'n siarad Cymraeg i weld meddyg Cymraeg os yw hynny'n bosibl heb ac am geisio sichrau bod meddygon teulu sy'n siarad Cymraeg ym mhob meddygfa a phwysigrwydd y drefn hyfforddi meddygon ar gyfer hynny.” “*…Reception workers have a lot of responsibility to make decisions on behalf of a patient in a way, and they need to be educated on the importance of giving a Welsh-speaking patient to see a Welsh doctor if that is possible, also trying to ensure that there are Welsh-speaking family doctors in every surgery and the importance of the doctor's training procedure for that.”* (Female, 40-49)
	9. Care should be offered in Welsh of the same standard as that of English	“Mae gweld ddiffyg y Gymraeg yn gofal iechyd yn anodd. Yn enwedig gyda'r henoed sydd yn defnyddio'r iaith yn dyddiol. Dyla'i fod ddisgwyl i'r gwasanaeth cael ei gynnal yn Gymraeg ir un safon a fydd yn Saesneg.” “*Seeing the lack of the Welsh language in healthcare is difficult. Especially with the elderly who use the language on a daily basis. It should be expected that the service is conducted in Welsh to the same standard as it will be in English.”* (Female, 20-29)
	10. Stigma and stereotyping	“Yn teimlo mor rhwystredig yn ein Meddygfa am fod agwedd llawer sy'n cael eu cyflogi yno mor negyddol ac anwybodus tuag at yr Iaith Gymraeg. Dim ymdrech gan lawer ohonynt i GEISIO dysgu'r Gymraeg a llawer wedi dod dros Glawdd Offa am fod tai yn rhatach yng Nghymru a byw yma, mewn gwlad wahanol, yng nghefngwlad, ar eu telerau hwy eu hunain ac agwedd fod pawb ymhobman yn gallu siarad Saesneg” “*Feeling so frustrated in our Surgery because the attitude of many who are employed there is so negative and ignorant towards the Welsh Language. Many of them make no effort to TRY to learn the Welsh language and many have come over Offa's Dyke because houses cheaper in Wales and live here, in a different country, in the country, on their own terms and with the attitude that everyone everywhere can speak English”* (Female, 70-79)
	11. No feelings of stigma	“Wedi cael gwasanaeth da gan feddyg teulu lleol oedd yn dysgu Cymraeg ac yn gallu siarad digon o Gymraeg i siarad gyda fy mhlentyn bach.” “*Had good service from a local family doctor who was learning Welsh and could speak enough Welsh to talk to my toddler.”* (Female, 40-49)

## Discussion

Effective communication is essential for quality healthcare, yet the impact of limited bilingual provision on first-language Welsh speakers remains underexplored. This mixed-methods study aimed to assess public awareness, experiences, and perceptions of bilingual care in primary healthcare settings.

Awareness of language rights was low, only a third of respondents were aware that WLS apply to hospitals but not most GPs. Given that the WLS were implemented in 2016, this suggests a slow dissemination of public information. Interestingly, awareness of language rights increased with age. Focus group comments (Table 2: A change in personal perspectives over time) suggested that younger generations often face social pressures at school, where English is the dominant social language, but perspectives can shift over time. Personal experiences such as caring for children or older relatives, or developing a stronger sense of identity, could heighten awareness. This is consistent with themes from the questionnaire's free-text comments. Growing awareness may prompt some to engage with regulations and policies which may have previously been overlooked. Overall, the lack of clarity regarding language rights in healthcare highlights the need for a Welsh Government public campaign across all age groups to improve understanding.

Participants from Welsh-speaking regions were more likely to perceive benefits to Welsh-language healthcare and actively choose GPs offering Welsh provision. While 84% preferred speaking Welsh with reception staff, Welsh provision is inconsistently promoted. Free-text comments highlighted feelings of stigmatization, injustice, and frustration (Table 3) with bilingual services resembling a “postcode lottery,” “*No one ever offers a Welsh language service. Sometimes it's available but not promoted.”* This observation raises concerns that services may exist but go underutilized due to a lack of active offer, promotion, or visibility. The *Iaith Gwaith* (Working Welsh) logo, launched in 2005 by the Welsh Language Commissioner, identifies Welsh-speaking staff. Its aim is to facilitate the normalization of the “active offer” of Welsh, making its availability visible, reducing stigma and awkwardness when requesting Welsh services. However, the logo's promotion and consistent use in GPs is limited, and it's meaning not widely understood. Greater awareness and consistent use of the *Iaith Gwaith* logo could empower patients to identify and request services in their preferred language.

Only 18% of respondents felt their need to use Welsh was taken seriously in healthcare settings. Some described relying on unofficial interpreters, for example, bilingual staff, family members or the need for “internal” translation (when switching from first to second language) (Table 3). Although no comments described health carers as translators, this is known to occur.^24^ One participant reported being questioned for translating for their child during a consultation, highlighting stigma, discomfort, and cultural insensitivity. Respondents also noted the presumption that English is the default language, despite Welsh-speaker rates of 52% to 76% in North and West Wales (ONS, 2021). The General Medical Council (2013) emphasizes that healthcare providers must communicate in a way patients understand, while translation is helpful,^24^ there is a risk of miscommunication, particularly when handling complex medical terms.^25^ Research supports the use of trained interpreters, but direct communication in a patient's first language remains safest. One participant also raised child-safety concerns when communication with a health visitor was not possible. Overall, these situations increase stress for both patients and their carers. For Welsh speakers, using their first language can improve communication, enhance comfort, clarity, and relief, particularly in vulnerable situations. This is consistent with international research showing that first-language care can foster trust, alleviate stress, and improve patient–physician relationships.^26,27^ This can lead to more accurate diagnoses and improved outcomes.^28^

About 32% from Welsh-speaking regions reported feeling restricted when unable to communicate in Welsh. This finding is consistent with other Welsh research, a pharmacy study reported 54% of respondents struggled to find English words for symptoms.^28^ Research also shows that Welsh speakers describe pain using unique terms and metaphors not captured by English-only tools like the McGill Pain Questionnaire.^29^ This highlights the need for culturally and linguistically appropriate assessment tools. Evidence suggests that language concordance may also improve physical health outcomes. For example, Diabetic Latino patients in the United States showed improved glycemic control when switching to Spanish-speaking physicians.^30^ Similarly, Welsh-speaking physiotherapy patients had better outcomes with bilingual therapists.^31^ These findings highlight that language use in healthcare affects both emotional wellbeing and clinical outcomes.

Respondents expressed dissatisfaction with having to forgo their right to use their preferred language for faster healthcare access. However, they also acknowledged NHS staffing challenges and a shortage of Welsh-speaking professionals. Recruiting and retaining GPs is particularly challenging in rural areas.^32^ Research also shows that although the WLS exist, Welsh is often missing from health boards’ well-being objectives, limiting their implementation.^33^ The Welsh Government's “*More Than Just Words”* strategy promotes an “active offer” approach, but more robust infrastructure and training are needed.^34^ Respondents supported recording their language preference in medical notes. However, one expressed concern that records must remain accessible to all doctors, regardless of language. There is indeed a need for guidance on safe communication in multilingual contexts. International models, such as those developed for Chinese patients in Australia^35^ and Euskara speakers in the Basque Country^36^ could inform future frameworks in Wales, as examples of how we might deliver healthcare in a language that is considered a minority in a mainly English or Spanish setting respectively.

Importantly, some bilingual individuals, such as people with dementia, may lose fluency in their second language,^37^ highlighting the need for language-sensitive assessments. While many Welsh speakers are fluent in English some may struggle to express themselves fully, particularly during emotionally charged or complex medical consultations.^15,29^ Participants’ comments revealed themes of frustration, unmet needs and emotional strain, particularly among vulnerable groups. These findings reflect tensions between language rights and service accessibility.

## Limitations and Conclusion

This questionnaire targeted Welsh speakers which may have introduced responder bias, as they may be more likely to engage with the topic. Nonetheless, capturing their experiences remains relevant and valuable. We also acknowledge that the questionnaire was not expert-reviewed and some wording could be considered “leading,” for example, “having to sacrifice their preferred language.” However, this reflects perceptions expressed by Welsh speakers, and this language was also used in the focus groups (Table 2: A patient's priorities/1. Quality of healthcare). The Welsh Government also recognizes the risks of inadequate first-language provision.^34^ Notably, several respondents expressed passion for the topic, emphasizing its importance and thanking us for conducting the research. Overall, our findings show that while Welsh speakers value bilingual healthcare, many are not offered the use of Welsh. The resulting disparity in service availability affects comfort, communication, and care quality. There is a need to improve awareness, enable active offers, and strengthen rights to support care in a patient's preferred language.

## Supplemental Material

sj-docx-1-jpx-10.1177_23743735261417165 - Supplemental material for Patient Experience and Perception of First Language Usage in Healthcare: The Welsh Perspective Supplemental material, sj-docx-1-jpx-10.1177_23743735261417165 for Patient Experience and Perception of First Language Usage in Healthcare: The Welsh Perspective by Maisie E. Edwards, MSc, Owen Bodger, PhD, Menna Brown, PhD, Llinos Roberts, MD, Luke D. Roberts, PhD, Jeffrey S. Davies, PhD, and Alwena H. Morgan, PhD in Journal of Patient Experience
