# Supplementary material for: Patient Experience and Perception of First Language Usage in Healthcare: The Welsh Perspective
Source: J Patient Exp. 2026 Feb 9;13:23743735261417165. doi: 10.1177/23743735261417165 (PMC12886726; doi:10.1177/23743735261417165)
Supplement: sj-docx-1-jpx-10.1177_23743735261417165 - Supplemental material for Patient Experience and Perception of First Language Usage in Healthcare: The Welsh Perspective [file sj-docx-1-jpx-10.1177_23743735261417165.docx]

Appendix 1

Questionnaire

1

**Beth yw eich oedran?** 
*How old are you?*

- <20
- 20-29
- 30-39
- 40-49
- 50-59
- 60-69
- 70-79
- 80-89
- >90

2

**Beth yw eich rhyw?** 
*What is your gender?*

- Benyw / *Female*
- Gwryw / *Male*
- Well gen i beidio â dweud / *I would rather not say*

3

**Ym mha sir ydych chi wedi byw yr hiraf?** 
*In which county have you lived the longest?*

- Ynys Môn / *Isle of Anglesey*
- Gwynedd
- Conwy
- Sir Ddinbych / *Denbighshire*
- Sir y Fflint / *Flintshire*
- Wrecsam / *Wrexham*
- Ceredigion
- Powys
- Sir Benfro / *Pembrokeshire*
- Sir Gaerfyrddin / *Carmarthenshire*
- Abertawe / *Swansea*
- Castell-Nedd Port Talbot / *Neath Port Talbot*
- Pen-y-bont ar Ogwr / *Bridgend*
- Bro Morgannwg / *Vale of Glamorgan*
- Rhondda Cynon Taf
- Caerdydd / *Cardiff*
- Merthyr Tudful / *Merthyr Tydfil*
- Caerffili / *Caerphilly*
- Casnewydd / *Newport*
- Torfaen
- Blaenau Gwent
- Sir Fynwy / *Monmouthshire*

4

**Beth yw’r lefel addysg uchaf yr ydych wedi’i gyflawni?** *What is the highest level of education you have achieved?*

- TGAU / Lefelau O // *GCSE / O-Levels*
- Lefelau A / Cymhwyster sy'n cyfateb i Lefelau A // *A Levels / Qualifications that correspond to A Levels*
- Gradd Israddedig / *Undergraduate Degree*
- Gradd Ol-raddedig / *Postgraduate Degree*
- Diploma Ôl-raddedig / *Postgraduate Diploma*
- Cymhwyster Galwedigaethol / *Vocational Qualification*
- HNC/HNV
- Cymhwyster Proffesiynol / *Professional Qualification*
- Arall / *Other* __________________________________________________
- Dim cymwysterau / *No qualifications*
- Well gen i beidio â dweud */ I would rather not say*

5

**Pa lefel o addysg trwy gyfrwng y Gymraeg ydych chi wedi ei dderbyn (unrhyw bwnc)?**
*What level of education through the medium of Welsh have you received (any subject)?*

- Cynradd / *Primary*
- Uwchradd (TGAU) / *Secondary (GCSE)*
- Uwchradd (Uwch Gyfrannol/Safon Uwch) / *Secondary (AS / A – Level)*
- Addysg Uwch yn y Brifysgol / *Higher Education in University*
- Dim addysg trwy gyfrwng y Gymraeg / *No education through the medium of Welsh*

6

**Beth yw eich statws gwaith cyfredol?** 
*What is your current work status?*

- Cyflogedig / Hunangyflogedig // *Employed / Self-employed*
- Wedi ymddeol / *Retired*
- Cyfnod Mamolaeth / *Maternity Leave*
- Ddim mewn gwaith am resymau penodol / *Not in work for specific reasons*
- Myfyriwr / *Student*
- Arall / *Other* __________________________________________________
- Well gen i beidio â dweud / *I would rather not say*

7

**Beth yw prif iaith eich aelwyd?** 
*What is your main language at home?*

- Cymraeg / *Welsh*
- Saesneg / *English*
- Y ddwy / *Both*
- Arall / *Other* __________________________________________________

8

**Ym mha iaith rydych mwyaf cyfforddus yn mynegi eich hun ar lafar?** 
 *In which language do you feel most comfortable expressing yourself verbally?*

- Cymraeg */ Welsh*
- Saesneg / *English*
- Y rhyddid i siarad Cymraeg a Saesneg / *The freedom to speak both Welsh and English*
- Arall / *Other* __________________________________________________

9
**Ym mha iaith rydych mwyaf cyfforddus yn ysgrifennu?** 
*In which language do you feel most comfortable writing?*

- Cymraeg / *Welsh*
- Saesneg / *English*
- Cyfforddus i ysgrifennu yn Gymraeg a Saesneg / *Comfortable writing in both Welsh and English*
- Arall / *Other* __________________________________________________

10

**Ym mha iaith rydych mwyaf cyfforddus yn darllen?** 
*In which language do you feel most comfortable reading?*

- Cymraeg / *Welsh*
- Saesneg / *English*
- Cyfforffus i ddarllen yn Gymraeg a Saesneg / *Comfortable to read in both Welsh and English*
- Arall / *Other*

11

**A oeddech chi'n ymwybodol nad ydych yn gallu hawlio gofal iechyd sylfaenol trwy gyfrwng y Gymraeg?** 
*Are you aware that you do not have the right to demand primary healthcare through the medium of Welsh?*

- Oeddwn */ I was aware*
- Nac oeddwn / *I wasn't aware*
- Heb ystyried / *I hadn't considered it*

12

**A oeddech yn ymwybodol bod yna ddisgwyliad i ysbytai gwrdd â’r safonau Cymraeg sy’n cael eu rhoi gan Gomisiynydd y Gymraeg (o dan yr hawliau at wasanaethau yn y Gymraeg) ond nad ydynt yn ddisgwyliedig ar gyfer sefydliadau gofal iechyd sylfaenol ee. Meddygfeydd.**
*Were you aware that hospitals are expected to meet Welsh language standards set by the Welsh Language Commissioner (under the rights to Welsh language services) but they are not expected of primary healthcare organizations e.g. GP surgeries.*

- Oeddwn / *I was aware*
- Nac oeddwn / *I wasn't aware*
- Heb ystyried / *I hadn't considered it*

13

**Ydych chi erioed wedi dewis meddygfa oherwydd bod ganddyn nhw feddyg sy'n siarad Cymraeg?** 
*Have you ever chosen a GP surgery based on the fact they have a Welsh speaking doctor?*

- Ydw / *Yes I have*
- Nac ydw / *No I haven't*

14

**Os ydych chi fel arfer yn cael eich ymgynghoriadau meddygon teulu yn Saesneg, a fyddech chi’n teimlo'n fwy cyfforddus pe byddent yn cael eu cynnal yn y Gymraeg / yn ddwyieithog?**
*If you usually have your GP consultations in English, would you feel more comfortable if they were held in Welsh/bilingual?*

- Llawer mwy cyfforddus / *Much more comfortable*
- Mwy cyfforddus/ *More comfortable*
- Ddim yn gyfforddus nac yn anghyfforffus / *Neither comfortable nor uncomfortable*
- Ddim llawer mwy cyfforddus / *Not much more comfortable*
- Ddim o gwbl / *Not at all*

15

**Ym mha iaith ydych chi’n dueddol o siarad gyda’ch meddyg teulu yn ystod ymgynghoriad clinigol, os ydynt yn gallu siarad Cymraeg?**
*In which language do you tend to speak to your GP during clinical consultations, if they are Welsh speaking?*

- Cymraeg / *Welsh*
- Saesneg / *English*
- Y ddau / *Both*
- Arall / *Other* __________________________________________________
- Nid yw fy meddyg yn siarad Cymraeg / *My doctor doesn't speak Welsh*

16

**Wrth wneud apwyntiad gyda’r meddyg teulu, ydych chi wedi gofyn am feddyg penodol oherwydd ei fod yn gallu siarad Cymraeg?**
*While making an appointment with the doctor, have you ever asked for a specific doctor because he/she speaks Welsh?*

- Ydw */ I have*
- Nac ydw */ I have not*

17

**Yn mha iaith y byddech yn teimlo mwyaf cyfforddus yn cyfathrebu gyda’r derbynnydd?**
In which language would you feel most comfortable communicating with the receptionist in?

- Cymraeg / *Welsh*
- Ddwyieithog / *Bilingually*
- Saesneg / *English*
- Arall / *Other* __________________________________________________

18

**Yn eich barn chi, ydy defnydd y Gymraeg wrth dderbyn gofal iechyd sylfaenol yn rhan bwysig o’ch gofal?**
*Do you think the use of Welsh language when receiving primary health care is an important part of your care?*

- Pwysig iawn / *Very important*
- Pwysig / *Important*
- Ddim yn bwysig / *Not important*
- Dim ots / *Don't mind*

19

**Ydych chi’n teimlo eich bod yn elwa o dderbyn dogfennau dwyieithog sy’n gysylltiedig gyda gofal iechyd sylfaenol?**
*Do you feel you benefit from receiving bilingual documents related to primary health care?*

- Ydw / *Yes*
- Nac ydw / *No*
- Ddim yn sicr / *Not sure*

20

**A fyddech yn elwa o ddarpariaeth gofal iechyd ar lafar yn y Gymraeg?**
*Would you benefit from verbal health care provision in Welsh?*

- Byddwn / *Yes*
- Na fyddwn / *No*
- Ddim yn sicr / *Not sure*

21

**Ydych chi erioed wedi cael cynnig gan eich meddyg teulu i ddefnyddio’r Gymraeg fel eich iaith ddewisol yn ystod ymgynghoriad?**
*Have you ever had an offer from your GP to use Welsh as your language of choice during consolation?*

- Ydw / *Yes*
- Nac ydw / *No*
- Ddim yn sicr / *Not sure*

22

**Ydych chi erioed wedi bod mewn sefyllfa ble roeddech yn teimlo'n rhwystredig wrth ond allu trafod yn Saesneg gyda'ch meddyg teulu?** 
*Have you ever been in a situation where you've felt restricted by being only able to discuss in English with your GP?*

- Ydw / *Yes*
- Nac ydw / *No*
- Ddim yn sicr / *Not sure*
- Sylwad / *Comment*

__________________________________________________

23

**Petaech wedi gallu siarad yn y Gymraeg ac felly yn teimlo'n fwy cyfforddus gyda'ch meddyg teulu, ydych chi'n credu y byddech wedi derbyn gwasanaeth gofal iechyd gwell?** 
*If you were able to speak in Welsh and therefore feel more comfortable with your GP, do you believe that would have received a better healthcare service?*

- Ydw / *Yes*
- Nac ydw / *No*
- Ddim yn sicr / *Not sure*
- Sylwad / *Comment*

__________________________________________________

24

**A fyddech yn hapus i nodi eich iaith ddewisol ar eich cofnodion meddygol?**
*Would you be happy to note your language preference on your medical records?*

- Hapus / *Happy*
- Dim ots / *Don't mind*
- Anhapus / *Unhappy*

25

**A ydych chi’n teimlo bod eich angen neu hawl i ddefnyddio’r Gymraeg (neu siarad yn ddwyieithog) yn ystod gofal iechyd sylfaenol yn cael ei gymryd o ddifri gan lefydd sy’n darparu gofal iechyd sylfaenol?**
*Do you feel that your need or right to use Welsh (or bilingually) during primary health care is taken seriously by places that provide primary health care?*

- Ydw / *Yes*
- Nac ydw / *No*
- Ddim yn sicr / *Not sure*

26

**Os taw'r Gymraeg neu iaith leiafrifol arall yw eich iaith ddewisol, ydych chi'n teimlo ei bod hi'n iawn eich bod yn gorfod aberthu'ch iaith ddewisol er mwyn cyrchu gwasanaeth gofal iechyd yn gynt?** 
*If Welsh or another minority language is your language of preference, do you feel that it's right you have to sacrifice your preferred language in order to access healthcare services more quickly?*

- Ydw / *Yes*
- Nac ydw / *No*
- Dim ots / *Don't mind*
- Dwi erioed wedi aberthu fy iaith ddewisol / *I have never sacrificed my preferred language*
- *Sylwad / Comment* __________________________________________________

27

**A oes unrhyw beth arall yr hoffech ei rannu am eich profiadau neu ddiffyg profiadau o defnyddio'r Gymraeg wrth gyrchu gofal iechyd sylfaenol?**
*Is there anything else you want to share about your experiences or lack of experiences of using the Welsh language while accessing primary health care?*

________________________________________________________________

________________________________________________________________

________________________________________________________________

________________________________________________________________

________________________________________________________________
